# How might we increase physical activity through dog walking?: A comprehensive review of dog walking correlates

**DOI:** 10.1186/1479-5868-11-83

**Published:** 2014-08-20

**Authors:** Carri Westgarth, Robert M Christley, Hayley E Christian

**Affiliations:** Department of Epidemiology and Population Health, Institute of Infection and Global Health, and School of Veterinary Science, Faculty of Health and Life Sciences, University of Liverpool, Leahurst Campus, Chester High Road, Neston, Cheshire CH64 7TE UK; NIHR Health Protection Research Unit in Emerging and Zoonotic Infections, Liverpool, L69 7BE UK; Centre for the Built Environment and Health, School of Population Health, and Telethon Kids Institute, The University of Western Australia (M707), 35 Stirling Highway, Crawley, WA 6009 Australia

**Keywords:** Dogs, Walking, Physical activity, Review, Public health, Epidemiology, Intervention studies

## Abstract

**Background:**

Physical inactivity and sedentary behaviour are major threats to population health. A considerable proportion of people own dogs, and there is good evidence that dog ownership is associated with higher levels of physical activity. However not all owners walk their dogs regularly. This paper comprehensively reviews the evidence for correlates of dog walking so that effective interventions may be designed to increase the physical activity of dog owners.

**Methods:**

Published findings from 1990–2012 in both the human and veterinary literature were collated and reviewed for evidence of factors associated with objective and self-reported measures of dog walking behaviour, or reported perceptions about dog walking. Study designs included cross-sectional observational, trials and qualitative interviews.

**Results:**

There is good evidence that the strength of the dog-owner relationship, through a sense of obligation to walk the dog, and the perceived support and motivation a dog provides for walking, is strongly associated with increased walking. The perceived exercise requirements of the dog may also be a modifiable point for intervention. In addition, access to suitable walking areas with dog supportive features that fulfil dog needs such as off-leash exercise, and that also encourage human social interaction, may be incentivising.

**Conclusion:**

Current evidence suggests that dog walking may be most effectively encouraged through targeting the dog-owner relationship and by providing dog-supportive physical environments. More research is required to investigate the influence of individual owner and dog factors on ‘intention’ to walk the dog as well as the influence of human social interaction whilst walking a dog. The effects of policy and cultural practices relating to dog ownership and walking should also be investigated. Future studies must be of a higher quality methodological design, including accounting for the effects of confounding between variables, and longitudinal designs and testing of interventions in a controlled design in order to infer causality.

## Background

Increasing evidence supports the public health value of pet ownership in western countries [1, 2]. The study of the relationship between dog ownership, dog walking and physical activity has recently received significant attention [3]. Many of these studies have highlighted that walking a dog could be a potentially important population-level strategy for increasing physical activity, particularly as they are present in 23% of UK [4], 36% of Australian [5] and 47% of USA households [6]. This is important because in many developed countries a large proportion of the population are not sufficiently active for health benefit [7–9]. For example, in the UK, only 39% of men and 29% of women meet the government’s physical activity recommendations [9] of 150 minutes of moderate-vigorous intensity physical activity per week (usually interpreted as at least 30 minutes of physical activity on at least on five days a week) [10]. If all dog owners (between 20-40% of the population) briskly walked their dogs for at least 30 minutes each day they would easily achieve the recommended level of physical activity.

There is considerable evidence that dog ownership is associated with higher levels of physical activity in adults. In a meta-analysis of studies conducted to date owners who walk their dogs walked a median duration of 160 minutes per week and a median frequency of four times per week [3]. Increased physical activity has also been observed in children who have a dog [11–13] but not conclusively in adolescents [14, 15]. A direction of causation has also been confirmed longitudinally by demonstration of an increase in walking on acquisition of a dog [16]. Further, the dog-owner relationship has the potential to enhance health to a greater degree than if walking alone or with a person. For example, walking with (vs. without) a dog has been shown to be a greater buffer against stress due to the positive effects on parasympathetic neural activity [17]. Dogs also appear to have a special ability in augmenting maintenance of physical activity over the longer term [18]. Finally, dog walking impacts both people and their pets. The exercise levels of dogs correlates well with their owners activity levels [19] and the exercise levels of dogs has been shown to be inversely associated with dog obesity, an increasing animal welfare issue [20–22].

**Figure 1:**
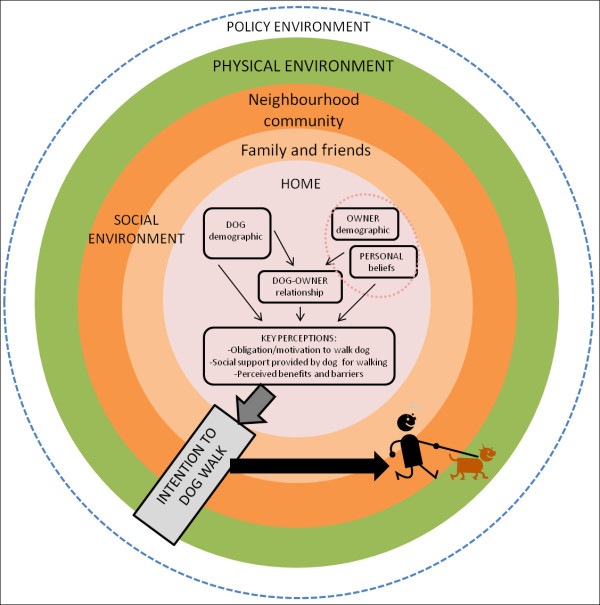
**Social-ecological model of the correlates of dog walking.**

However, a large proportion of the community who own a dog do not walk it or do so only occasionally, despite most dog owners believing that exercising their dog regularly is good for the human-animal relationship and good for the animal’s health [23]. It is estimated that only 60% of dog owners walk their dog at all [3]. Owners who do not walk their dogs regularly are less likely to achieve the recommended physical activity levels compared with dog walkers [24–27], and people without a dog [28]. Furthermore, in the US there is some evidence to suggest that dog walking is decreasing in popularity as a means of physical activity [29]. Considering a significant proportion of households own dogs and many do not walk their dog regularly dog owners should be targeted in physical activity interventions. Effective strategies aimed at increasing dog owners’ walking levels requires a better understanding of the correlates of dog walking [3]. Although a relatively new area of research a number of studies have examined the correlates of dog ownership, dog walking and physical activity, however to date this evidence has not been comprehensively reviewed. As dog walking is a specific behaviour different to general physical activity or even other types of walking, it requires a context specific approach to examining these correlates [30]. Using a social-ecological approach [31] we have reviewed the evidence and through this process developed a model of the physical-environment, social-environment, personal and dog-related factors associated with dog walking (Figure 1). This paper uses this model as a framework for presenting the results of our review.

## Aim

Thus, the aim of this paper is to comprehensively review and summarise the evidence of the correlates of pet dog walking, to provide evidence to guide future physical activity intervention research and programs involving dog owners.

## Methods

**Figure 2:**
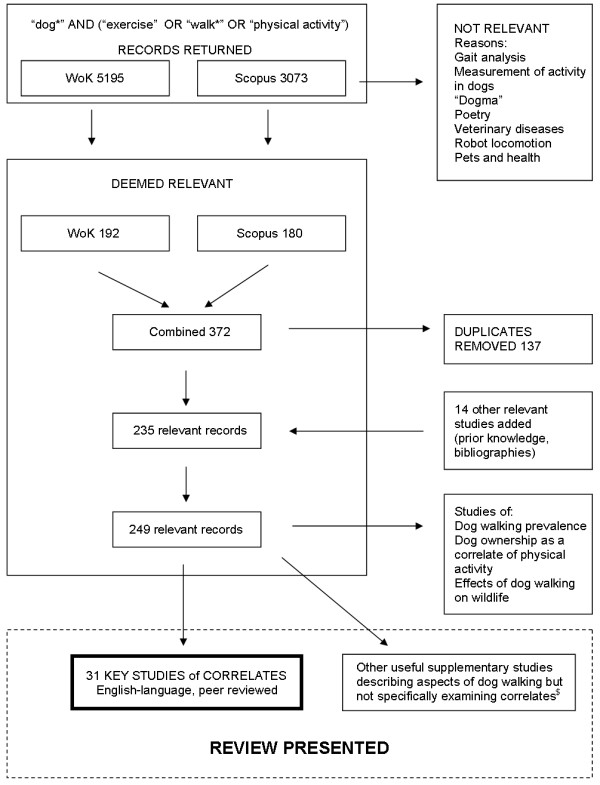
**Literature search strategy for review of the evidence of the correlates of dog walking.** Legend: ^$^ = studies specifically concerning dog obesity/weight status alone have been reported elsewhere so were excluded from the key studies review and treated as supplementary information. WoK = Web of Knowledge.

A review of all published studies from 1990-October 2012 was conducted by the primary author (CW) and corroborated by a second author (HC), using database searches of Web of Knowledge (WoK) and Scopus. The search terms were developed to be sensitive and non-specific, and were carried out in the format of [“Dog*” and (“exercise” or “walk*” or “physical activity”)] in the title, abstract, or keywords. In addition, other relevant studies were identified from prior expert knowledge and searching of bibliographies. The full search strategy is displayed in Figure 2. In order to maximise the evidence gathered we took the novel approach of including both the human health literature and the veterinary literature.

Thirty-one key studies were identified (Table 1). Due to this being a relatively new area of research, most of the studies (apart from one small pilot randomised-controlled trial) were observational cross-sectional questionnaire surveys, or qualitative interviews. Thus, the studies were not rated further for quality of methodological design as these are already considered low or very low levels of evidence by existing systematic review hierarchy of evidence grading systems [32] (e.g. level 4 of the Centre for Evidence-based Medicine grading [33]). Further methodological issues observed included small sample sizes, use of convenience samples with a high likelihood of bias, and incomplete reporting of non-significant findings. In particular, sample sizes and the use of simple univariable associations or multivariable analysis adjusting for confounding variables is noted in our results tables, as these greatly affects the strength of the evidence and should be noted when interpreting individual study findings. In addition, studies reported a number of different dog walking relevant outcomes, and considered factors measured in different ways, thus, meta-analysis was not performed.

**Table 1:** **Summary of studies of the correlates of dog walking**

Author	Study type	N	Location	Outcomes studied	Confounding considered?
		Owner		Owner PA	
Oka and Shibata, 2012 [27]	Cross-sectional survey	930 adult DO	Japan	DW yes/no	Yes
Rohlf et al., 2012 [23]	Cross-sectional survey (non-significant variables in model unknown)	1016 adult DO	Australia	Dog exercise frequency	Yes
Rhodes et al., 2012 [41]	Randomised Controlled Trial	58 inactive DO adults	Canada	DW min/week and pedometer step count	Yes
Degeling et al., 2012 [40]	Cross-sectional survey	241 DO	Canada	DW frequency, achieving 150 m/week	Yes
Arnberger and Eder, 2012 [52]	Cross-sectional survey of visitors	330 visitors	Austria	Perceptions of parks and coping behaviours	No
Ioja et al., 2011 [56]	Park observation and visitor perception survey	5240 DO and NDO adults	Romania	Frequency and length of visits plus perceptions of parks	No
Heuberger and Wakshlag, 2011 [36]	Cross-sectional survey	318 DO adults	USA	Dogs activity, owner’s exercise	No
McCormack et al., 2011 [58]	Cross-sectional survey	506 DO adults	Canada	DW yes/no, DW frequency	Yes
Scheibeck et al., 2011 [60]	Qualitative interview	23 elderly DO	Austria	DW frequency and distance	No
Hoerster et al., 2011 [26]	Cross-sectional survey (some non-significant variables unknown)	984 adult DO	USA	DW yes/no	Yes
Christian et al., 2010 [25]	Cross-sectional survey	483 adult DW	Australia	Regular/irregular DW	Yes
Rohlf et al., 2010 [43]	Cross-sectional survey of exercise intentions (non-significant variables in model unknown)	182 adult DO	Australia	Dog exercise intentions, dog exercise amount (frequency and duration)	Yes
Westgarth et al., 2009 [34]	Cross-sectional survey	224 DO households	UK	Number of areas visited on dog walks	No
Lee et al., 2009 [57]	‘Dog park’ observation and user perception survey ‘Dog park’ observation and user perception survey	267 DO who use dog parks	USA	Perceptions of dog park use	No
Coleman et al., 2008 [59]	Cross-sectional survey	2199 adult DO and NDO	USA	DW yes/no	No
Cutt et al., 2008a [16]	Qualitative focus groups	51 adult DO	Australia	Barriers and motivators to DW	No
Knight and Edwards, 2008 [47]	Qualitative focus groups	65 adult DW	UK	Exploring aspects of DO and DW	No
Cutt et al., 2008b [24]	Cross-sectional survey	629 adult DO	Australia	DW yes/no	Yes
Thorpe et al., 2006 [83]	Cross-sectional survey	394 DO	USA	DW yes/no	No
Ham and Epping, 2006 [101]	Cross-sectional survey	1282 DW youth and adults	USA	DW frequency and duration	No
Brown and Rhodes, 2006 [42]	Cross-sectional survey	351 adult NDO and DO	Canada	Walk frequency and duration	No
Schofield et al., 2005 [102]	Cross-sectional survey	1237 adult NDO and DO	Australia	Walking for leisure	No
Suminski et al., 2005 [48]	Cross-sectional survey	474 adult NDO and DO	USA	DW yes/no	Yes
Carver et al., 2005 [49]	Cross-sectional survey (non-significant variables not presented)	347 adolescents	Australia	DW yes/no weekends	Yes
Arnberger and Hinterberger, 2003 [51]	Observations of park use and cross-sectional survey	140 DW 420 NDW interviewed	Austria	Frequency and duration of park visit	No
		**Dog**		**Dog PA**	
Westgarth et al., 2008 [35]	Cross-sectional survey	279 dogs	UK	Walk frequency and duration	No
Tami et al., 2008 [37]	Cross-sectional survey	181 dogs (Argentine Dogos)	Italy	Daily walk duration	No
Masters and McGreevy, 2008 [103]	Cross-sectional survey (non-significant findings not presented)	690 dogs	Australia	Walk frequency	No
Robertson, 2003 [20]	Cross-sectional survey	860 dogs	Australia	Land exercise yes/no	No
Kobelt et al., 2003 [39]	Cross-sectional survey	254 dogs	Australia	Walk frequency	No
Podberscek and Serpell, 1997 [38]	Cross-sectional survey	435 dogs (English Cocker Spaniels)	UK	Frequency and duration of walks/exercise	No

PA = Physical activity, DO = Dog ownership or dog owner, NDO = Non-dog owner, DW = Dog walking or dog walker (owner who walks their dog), NDW = Non-dog walker (owner who does not walk their dog).

Here we present a review of the findings from the 31 key studies identified, plus relevant supplementary information to aid in understanding and depth of evidence. Variation in quality of evidence is also reflected in our judgment of the evidence presented. Studies investigated various outcomes relevant to dog walking, for example: frequency, duration, intention to dog walk, perceptions about dog walking, regular over rare dog walking, and any dog walking over none. Most studies recruited dog owners and analyses were performed at the owner level, although some studies from the veterinary literature recruited dogs and were analysed at the level of the dog (n = 6). For these we assumed that the dog was being walked by the owner rather than an external person and thus for some studies the owner’s self-report of the dog’s physical activity was used for our purposes as a proxy for the owner’s physical activity undertaken with their dog.

In accordance with our proposed model of dog walking behaviour, evidence of the correlates of dog walking were grouped into four categories of; dog-related (Table 2), social environment (Table 3), physical environment (Table 4), and owner-related (Table 5). Numbers presented in the tables refer to citations in the reference list.

**Table 2:** **Evidence of the DOG-RELATED (demographic and dog-owner relationship) correlates of dog walking**

		Evidence of a positive association	Evidence of a negative association	No evidence of an association	Where some elements show evidence of a positive association but others no evidence of an association
**Dog demographic**	Number of dogs	34	16*, 103	**40**, **26**, **25, 24**	
	Dog size	35, 102		**27**, 34, **24**, 42, 39	
	Dog type/breed	34		20	
	Dogs age		36, 35, 20	**26**, 34	16*
	Dog sex			34, 20	
	Dog neutered	103		34, 20	
	Dogs health/ability	16*		**26**, **24**	
	Dog weight status**		20	**25**, **24**	
	Dog behaviour problems		37, 38, 39	**24**, **26**	
	Perceived exercise requirements	16*, **23**, **40**, 41		**26**	
**Dog-owner relationship**	Dog attachment/interaction	**23**, **27**		**24**	
	Dog encourages/supports walking +companionship	16*, **24, 25**, **26**			
	Dog obligation	16*, **26**, 42			
	Feelings of guilt	16*			
	Perceived benefits dog health	16*, 41		**24, 25**, **26**	
	Perceived benefits dog behaviour	16*		**24**, **25**	
	Valuation of exercise for dog	41, **43**			
	Knowing dog enjoys going for a walk			**24, 25**	

*qualitative evidence. For quantitative observational studies, bolded citation number = multivariable adjusted evidence or unbolded citation number = univariable evidence. **Studies specifically concerning dog obesity/weight status alone have been reported elsewhere so were excluded from the key studies review and treated as supplementary information.

**Table 3:** **Evidence of the SOCIAL ENVIRONMENT correlates of dog walking**

	Evidence of a positive association	Evidence of a negative association	No evidence of an association
Perception that DW promotes social engagement	16*, 47*		**26**
Subjective norm of significant others about dog walking	**23**, **24**, 42		**25**
Other people’s dogs (e.g. large, uncontrolled, roaming, untrained, off leash, aggressive, fear)		16*, **49**	
Other dog owners not picking up after dog		16*	
Safety concerns		47*, **48, 49**	**24**, **26**
DW perceived as a deterrent for local crime/increasing feelings of safety	16*, 47*		
Conflict with other users of DW areas		16*	
Crowding in DW areas		51, 52*	

DW=Dog walking. *qualitative evidence. For quantitative observational studies, bolded citation number = multivariable adjusted evidence or unbolded citation number = univariable evidence.

**Table 4:** **Evidence of the PHYSICAL ENVIRONMENT correlates of dog walking**

	Evidence of a positive association	Evidence of a negative association	No evidence of an association
Accessibility/proximity to walking areas (particularly off-leash)	56*, **25, 43**, 57, 16*, **49**, 51	**58**	**24**, **48**
Indicate take dog to ‘dog park’ specific area			**26**
Park aesthetics/footpath provision/size/lighting/fencing	16*, 56*		
Dog-supportive features/enrichments and separate children’s play area	16*, **25**, 56*		**24**, **58**
Neighbourhood street pattern			**48**
Neighbourhood aesthetics			**26**, **48**
Neighbourhood walkability	59		
Urbanicity			101
Type of residence (attached vs separate)	**40, 58**, 60*		**27**
Backyard size		16*	39
Weather/temperature		57*	51

*qualitative evidence. For quantitative observational studies, bolded citation number = multivariable adjusted evidence or unbolded citation number = univariable evidence.

**Table 5:** **Evidence of the OWNER RELATED (Demographic and personal beliefs) correlates of dog walking**

		Evidence of a positive association	Evidence of a negative association	No evidence of an association	Where some elements show evidence of a positive association but others no evidence of an association
**Demographics**	Gender (female)	**40**, **58**		**27**, **26**, 59, 83, 101, **48**	
	Age (middle aged sometimes most likely)	**23**, **58**, 101		**27**, **40**, 34, 59**, 48**	
	Ethnicity (white)	59, 83			
	Education	**58**, 83		**27**, **40**, 59, **48**	
	Employment			**27**, 34	
	Income	**27**, **40**, **26**, 101		59	
	Health			**58**, 83	
	Weight status		**58**, 59, 83		
	Other people in household or dependents living at home	**58**		**27**, 34, 83	
	Marital status	**27**			
	Others in household walk dog			**24, 25**, **26**	
**Personal beliefs**	Theory of Planned Behaviour constructs	**26**, **43**			42
	Lack of time		**43**, 57*		

For quantitative observational studies, bolded citation number = multivariable adjusted evidence or unbolded citation number = univariable evidence.

## Review of the evidence of the correlates of dog walking

### Dog-related factors: Dog demographics

There was mixed evidence of an association between the number of dogs, size, type/breed, neuter status, health/ability, or weight status and dog walking (Table 2). Although it is already known that dogs that are overweight are exercised less frequently [20–22], two key studies found no evidence of an association between dog weight status and owner’s physical activity via dog walking after adjustment for other factors [24, 25]. There was no evidence of a relationship between sex of the dog and dog walking behaviour [20, 34]. Although some studies did not identify an association between dog age and walking [26, 34], there were a considerable number that suggested a negative association, that is younger dogs were walked more frequently and for longer periods [16, 20, 35, 36]. However the complexity of age as a factor is illustrated by the suggestion that young puppies also require less walks [16].

The evidence suggests that dog walking is associated with fewer dog behaviour related problems. Owners perceive that regular dog exercise is good for dog behaviours [23] such as destruction and barking [16]. Moreover, the amount that dogs are walked is negatively associated with their fears of strangers, noises and stimuli [37], exhibiting aggression [38], lack of obedience [37], pulling on the lead [38], and excessive barking, activity or escaping [39]. However, two studies of owner’s dog walking behaviour found no association between dog behavioural problems and dog walking [24, 26].

Finally, there appears to be sufficient evidence of a positive association between the perceived exercise requirements of dogs and dog walking. Dogs that are thought to require considerable exercise are walked more frequently and their owners are more likely to achieve 150 minutes per week of dog walking [40]. Moreover, owners that believe that all dogs should be exercised regularly are more likely to report that their dog receives ‘adequate’ amounts of exercise and also report exercising them more often [23]. However, one study showed no evidence of an association between perceived exercise requirement of the dog and dog walking after adjustment for other factors [26]. Pilot intervention studies may be targeting the perceived exercise requirements of the dog (through persuasive material of the benefits of exercise on canine health) with some success, with the intervention group achieving higher step counts (1823 daily steps) than the control [41]. However, both the intervention and control group were observed to increase their physical activity in this study.

### Dog-related factors: The dog-owner relationship

The dog-owner relationship appears to be the most important correlate of dog walking behaviour (Table 2). The dog-owner relationship includes factors such as attachment, frequency of interactions, feelings of support and motivation provided by the dog for walking and feelings of obligation towards walking the dog. For example, Japanese dog walkers had higher levels of attachment to their dogs than owners who did not walk their dog (OR =2.32 ‘high’ compared to ‘low’ attachment) [27] and in a sample of Australian owners and dogs, higher scores on a dog-owner interaction scale was associated with more frequent exercise [23]. Moreover, dog owners who perceive that their dog provides motivation, companionship and/or support for walking [16, 24, 25], or similarly, feel that the dog provides encouragement to walk [26], are more likely to walk the dog, or walk regularly with the dog. For example, not walking the dog was been shown to be significantly more likely in owners who do not perceive that their dog provides motivation (OR = 9.60) or social support (OR = 10.84) to walk, independent of other well-known correlates of physical activity. Notably, ‘knowing that the dog enjoys going for a walk’ does not appear to be associated with dog walking when support/motivation provided by the dog is already accounted for [24, 25].

‘Dog obligation’ is an owner’s sense of obligation and/or responsibility to walk their dog regularly and a feeling that their dog pressures them to take it for a walk [42] and has been shown to mediate the relationship between dog ownership and physical activity [26, 42]. It might also encompass valuing exercise for the dog [43] and ‘feelings of guilt’ [16], and is likely to be related to the strength of the dog-owner relationship/attachment. As mentioned previously, pilot intervention studies suggest that targeting the canine need for exercise, rather than the human need, through ‘persuasive material regarding canine health benefits’, may be a helpful approach for increasing owner activity [41]. Other research outside the key studies of dog walking correlates also confirms the central motivational role for walking as an obligation to support the needs of the dog; a commonly stated reason for adherence to a loaned dog walking programme for the elderly was that the dogs “need us to walk them” [44] and owners have been observed to talk to and make reference to their dogs wishes or needs whilst walking [45]. Moreover, a study of an owner-pet combined weight loss program described dogs as having three distinct (from human support) positive influences of being a consistent initiator of exercise (seeking out the owner for exercise), providing enjoyment of exercise (loving walking the dog), and engendering parental pride (doing something good for the dog) [46].

### Social environment factors

There was mixed evidence surrounding the motivating aspects of social facilitation that occurs during dog walking (Table 3). Although seeing other people walking their dogs has been suggested as a motivator [16, 47], when investigated quantitatively, there was no evidence of an association between the perception that dog walking promotes social engagement and dog walking [26]. Three studies support the positive influence of the subjective norm of significant others in relation to dog walking [23, 24, 42] with one study showing no association [25].

General safety concerns may be negatively associated with dog walking in females but not males [47–49]. However, two studies have shown that walking with a dog compared to without can also increase feelings of safety in the neighbourhood [16, 47] and other studies have found no association between safety and dog walking [24, 26].

Finally, social environment factors associated with the dog walking experience have been investigated. Concerns about dogs coming into contact with children or other park users and the potential for problems has been identified as a barrier to dog walking [16]. Moreover, crowded environments can discourage some dog walkers as they feel it interferes with walking their dog unleashed [50–52]; and they may put their dog on a leash or go home earlier than planned [52]. The evidence also suggests that factors related to other people’s dogs are negatively associated with dog walking. Two studies show that fear of coming into contact with other dogs is a barrier, in particular fear of large dogs, small feisty dogs, certain breeds, off-leash, uncontrolled, untrained or roaming dogs, and owners not adequately picking up after their dogs [16, 49].

### Physical environment factors

Access to, and quality of, dog-supportive features of parks were positively associated with dog walking (Table 4). Both key and supplementary studies show that being able to walk their dog off-leash is important for many dog owners [16, 53–55]. Accessible public open space for dogs and the provision of dog-related infrastructure within walking areas are also important to dog owners (e.g. clear signage, dog litter bags and bins, accessible water sources, fencing around designated off-leash areas, separation from children’s play areas, dog agility equipment, parks not being located near to busy roads and being well-fenced) [16, 56]. For example, owners who have access to a dog-supportive park within their neighbourhood are more likely to walk their dog regularly compared to rarely [25]. Furthermore, lack of access to appropriate walk areas is associated with reduced ‘intention’ to walk the dog [43]. However, owners who take their dog to a specifically designated ‘dog park’ (see [57]) are no more or less likely to dog walk than those who do not [26]. There is also evidence that owners who live within 1.6 km of a park with off-leash areas may be least likely to participate in dog walking at all [24, 58] but if they do walk with their dog the frequency [58] and the minutes spent [25] dog walking is higher.

There is also evidence that many of the physical environmental barriers and facilitators to general walking appear to also affect dog owners (e.g. park attractiveness, good lighting, footpath connectivity, wide footpaths on both sides of the road) [16, 56]. For example, dog walkers are more likely to live in high walkable neighbourhoods [59] and parents of children who walk dogs report better access to areas for exercise in their neighbourhoods than do parent of children who do not walk dogs [49]. However, other studies show no evidence of an association between neighbourhood aesthetics or street pattern [26, 48] and dog walking behaviour. Whilst only one study has investigated the association between urbanisation and dog walking there is sufficient evidence of a positive association between living in attached housing compared with detached and dog walking frequency [40, 58] although this association was not supported in a Japanese study [27]. The positive association between living in attached housing and dog walking is likely due to the need for short toileting walks when there is no garden or yard available. In elderly owners longer daily duration and distance of dog walking was reported for those who did not have a backyard [60]. A reduction in the size of backyards over time has also been suggested as a motivator for people to walk their dogs more often [16]; however in the one study conducted so far, there was no association between yard size and dog walk frequency [39]. Overall, it appears that the relationship between features of the physical environment and dog walking behaviour varies according to the study country of origin and thus is influenced by significant variations in urbanicity and cultural differences.

Finally, there has been little empirical research of the effect of weather or season on dog walking. The few findings from these key studies combined with other related research suggests that dog walkers at least appear to be less dependent than other walkers on variables such as the weather [61, 62], temperature [51], time of the year/season [51, 63] or the day of the week [64]. However, hot weather has been identified as a constraint in visiting a dog park [57].

### Owner-related factors: demographics

There was mixed evidence of an association between dog walking and owners’ gender, age, education, and income (Table 5). Moreover, there appears to be no evidence of an association between employment status and dog walking [27, 34]. There was also mixed evidence that living with other people or having dependents is associated with dog walking, limited evidence for marital status and there was no evidence that other members in the household walking the dog affects a person’s dog walking activity (Table 5). In the US, owners who walk their dogs are more likely to be white than other ethnicities [59, 65]. There is evidence that obese owners are less likely to walk their dog than healthy weight owners [58, 59, 65], but walking the dog does not appear to be associated with general health [58, 65].

### Owner-related factors: personal beliefs

Some studies have used health behaviour theoretical models such as the Theory of Planned Behaviour (TPB) [66] to guide their understanding of dog walking behaviour and ‘intention’ to walk a dog. For example, dog-walking self-efficacy is positively associated with dog walking [26] (Table 5). ‘Intention’ to exercise the dog predicts dog walking behaviour and there is evidence that this intention is itself influenced by various factors, including valuation of exercise, lack of time, dislike of exercise/affective attitude, instrumental attitude, subjective norm and how in control of the behaviour of dog walking owners feel (perceived behavioural control) [42, 43]. However the evidence is mixed for perceived behavioural control as one study found evidence of an association with intention [43] but the other no evidence [42]. In terms of perceived barriers to dog walking, lack of time has been perceived as a significant barrier [57] with dog walks being significantly longer in duration at weekends than on weekdays [51, 67]. Finally, greater levels of thought given before acquiring a dog is associated with owners reporting more frequent exercise of their dog [23].

## Discussion

The investigation of dog walking is an emerging and rapidly growing area of research in the context of an obesity epidemic and the need to find cost-effective strategies for increasing population levels of physical activity. This paper provides evidence of a number of different correlates specific to dog walking behaviour, including demographic factors related to the dog and owner, physical and social environmental factors, and less easily measurable aspects of the dog-human relationship. Overall, the evidence currently suggests that dog walking may be most effectively encouraged through: 1) targeting the dog-owner relationship to increase the sense of obligation to walk the dog as well as the emotional support the dog can provide to the owner; and 2) by the provision of dog-supportive physical environments. These contexts may be the best-buy strategies for future testing of health interventions to increase dog walking amongst dog owners. They may even require implementation together thus acting at multiple levels (both individual and environmental) for the most effective population change [68]. However, the quality of the evidence varies, with a number of different outcomes presented and various methodological approaches that may or may not adjust for the effect of confounding factors. Only one study used a randomised controlled trial design, thus potentially providing a stronger level of evidence of a causal mechanism for targeting the perceived canine need for exercise.

A key dog-related factor to encourage dog walking appears to be the strength of the relationship with the dog, in providing support for enjoyment for walking and a sense of obligation to walk the dog. Although there is evidence for other dog-related factors, studies suggest these disappear after adjusting for the support and motivation provided by the dog for walking [24, 25] or the sense of obligation to walk the dog [26, 42]. However it would be wrong to dismiss dog-related elements as not important; it is likely that dog demographic and behavioural factors contribute to intention to walk and practicalities of walking the dog, that drive the sense of obligation and feelings of support and motivation that arise. For example, these may be stronger for larger dogs than smaller because of differences in perceived or actual exercise needs. More research is required to better understand the correlates of dog walking behaviour, in particular the perception of the amount of exercise that different dogs require. For example, dog owners report that their dog receives ‘adequate’ exercise yet the interpretation of this appears to vary widely as they also report exercising their dogs 0 to >7 times a week [23]. This may provide scope for interventions that change the perception of what a dog needs in terms of exercise and requires further investigation for which in-depth qualitative enquiry may be useful.

The evidence also suggests that interventions that strengthen the relationship between the dog and their owner may be a useful strategy; doing obedience training or simply spending time with a dog can improve the relationship with the dog and the obedience of the dog [69]. However, it is unclear whether walking with a dog leads to a stronger relationship between the owner and their dog or if an existing strong relationship leads to more walking. Likewise, behavioural issues related to the dog (e.g., aggression or fears) may result in less owner physical activity because of decreased motivation to take the dog out in a public place, but may also be caused by it, as inadequate early experiences and socialisation in dogs can lead to the development of behavioural problems [70].

The design of areas intended for dog walking and how they fulfil dog and owner needs may be an important consideration for future interventions. In order to encourage more dog owners to walk their dogs the recreational areas used for dog walking must be both pleasurable and accessible, as opposed to the common phenomenon of relegating dog access only to the few areas left after other user types have been accommodated [71]. However, it is difficult to tease out cause and effect – do regular dog walkers choose to live closer to high quality parks where they can walk their dog or does living next to a high quality park cause people to walk their dog more? In particular there must be sufficient provision for off-leash walking as this appears to be important to owners; it is perceived that quality of life of the dog would be compromised if dogs could not be walked off-leash in areas [54]. This suggests that an important function of dog walking, particularly off-leash, is enhancing dog quality of life, and thus how a public space fulfils their dogs needs is important to owners.

We also know that walking with a dog is not simply a vehicle for physical activity; it also increases the frequency of interactions with people, especially strangers [72]. However, the effect appears to be dog specific, with breed and age of dog influencing these interactions [73]. In a UK study, 92% of owners noticed seeing the same people and their dogs regularly while walking their dog [35]. In the US, ‘dog parks’ are perceived as providing opportunity to meet neighbours and build community [57]. Pet ownership has also been positively associated with perceptions of neighbourhood friendliness, with pet owners score higher on social capital and civic engagement scales [74]. Dog walking is also a way to spend time with friends; in a UK study 38% of owners reported never walking their dogs with a group of friends and their dogs, but 3% did this every day [35]. If facilitation of social interactions is a potentially strong feature of dog walking practices, there may be a need to acknowledge and encourage this human need when designing interventions and space for dog walking, especially for those owners for which dog walking is a rare opportunity for social interaction (e.g., older adults). It is possible that health promotion activities focussed on increasing owner awareness of the importance to do regular physical activity may sometimes be misplaced; if the physical activity resulting from walking a dog is a secondary outcome to fulfilling other needs of the owner or dog. Owners may be more likely to participate in and sustain physical activity if they actually enjoy it, or feel that it benefits their dog, than if they are simply trying to be healthier and more active for themselves.

This review highlights a number of methodological aspects that have implications for future research and for the interpretation of our findings. The majority of studies to date have been cross-sectional in design which limits the ability to confirm the causal relationships between dog, owner, social and physical environment related factors and dog walking behaviour. Furthermore, the majority of studies did not use objective measures of physical activity. However there is some evidence to suggest that objective measurements of dog walking physical activity (i.e., accelerometers) correlate with self-report measures [59].

Moreover, many studies did not adjust for the effects of confounding variables. This means that reported findings for some factors may be due to their own correlations with other factors. For example, it is important to control for dog-related demographic variables such as dog size when considering the effects of dog behaviour, and vice versa. Furthermore, the type of dog and way it behaves may also be correlated with the owner-dog relationship. This may explain the sometimes conflicting findings for some of the correlates of dog walking reviewed, for example where sometimes dog behaviour or size appears to have an effect, but not in other studies where an aspect of the relationship or support/motivation provided by the dog is also examined. Furthermore, clear evidence regarding dog-related factors (size, breed, etc.) may also be difficult to gather because they are hard to accurately measure in self-completed surveys and there may not be sufficient power to detect differences, especially in the often small sample sizes used. It is also possible that interactions between dog age and breed as well as a non-linear relationship between dog age and dog walking behaviour may exist and this should be considered in future studies. Few studies in this review sought to adjust for well-known correlates of general physical activity, such as: socio-demographic (gender, age, country of origin, education, occupation, children at home <18 years); perceived physical–environmental; and family social support and intrapersonal factors [24]. Even where multivariable analyses were performed, some adjusted for a wide range of non-significant factors due to their theoretical importance and high face validity (e.g. [24, 25, 58]), whereas others did not clearly report adjustment for other factors (e.g. [23, 42]). Dog walking behaviour outcomes also varied widely between studies with some at the level of the dog and others at the level of the owner (see Table 1).

Finally, future research should include context-specific measures of both the dependent and independent variables [75]. For example, context-specific measures of intrapersonal factors such as ‘intention to walk the dog’ as opposed to measuring ‘intention to walk’ in general (e.g. [42]) may better capture the factors associated with dog walking behaviour. In addition, it is unclear whether correlates influence dog walking behaviour and/or if they effect intention to walk the dog. As highlighted in our proposed theoretical model (Figure 1) future research should examine the pathways through which the different correlates influence dog walking behaviour. A survey tool has been designed specifically for this type of research (the Dogs And Physical Activity Tool; DAPA Tool) [76]. The DAPA Tool has been shown to be a reliable tool for measuring important attributes and scales relating to dog owners’ physical activity behaviour and the context-specific factors that affect owners’ walking with their dogs. Future studies would be more easily comparable if consistent data collection methods were used, however, the DAPA Tool is likely to require further development for effective use in different contexts and cultural locations than its original design (for example to study children or other cultures than Australia where it was developed).

This review highlights that there has been little explicit research as to what dog walking actually is, to both the owner and the dog; what actually happens on a ‘dog walk’ and what functions it performs. It is recommended that physical activity behaviours are considered separately, in order to study and implement the most effective strategies to fit this particular physical activity context [30]. Previously, dog walking has been considered a leisure-time or recreational physical activity behaviour (e.g., [16, 63, 77–82]); non-exercise related walking [83]; chores/errands [84]; and even commuting physical activity [85]. Thus we recommend that in future dog walking is considered in its own right. The dog walking experience also depends on whether the dog is on or off-leash. Most dogs stay fairly close to their owners [35, 54] and a large proportion of the walk is spent sniffing, especially when off leash or if the dog is a ‘gundog’ type [86]. Further research is required to better understand the dog walking experience: its intended purpose (for recreation or transport or just a chore that has to be done); the intensity (light, moderate or vigorous) and pattern (long bouts or numerous short bouts) of the physical activity undertaken with a dog; and how this is affected by factors such as how the dog behaves; which in turn is affected by dog breed, whether the dog is on or off leash as well as the environment the dog is being walked in. Finally, while dog walking has the potential to facilitate increased physical activity in owners, forcing dogs to exercise with us may also raise ethical questions [87] for example if there is a mismatch between the needs of the dog and the wishes of the owner.

This emerging area of research is also affected by a frequent problem in sociological research; that people, including researchers, already have ‘common sense’ knowledge of what dog walking is to them. However, it cannot be assumed that this is the same across society; there is unlikely to be a single ‘type’ of dog walker and there are likely to be cultural differences even between western countries for example, the use of official ‘dog parks’ (in the USA but not the UK) where owners passively sit or stand is common [57]. Further research in different countries and cultures is required to target the benefits to be gained from dog walking, as multiple strategies are likely to be required; for example, initiation of dog walking, maintenance, or increasing frequency may all benefit from different strategies. Further qualitative and observational research is required to enhance understanding of the phenomenon and its complexity, in particular surrounding the ‘intensity’ of physical activity that occurs during ‘a dog walk’ and the everyday barriers that affect dog owners regardless of their best intentions to walk more. Much of the research to date has focused on why people do walk their dog rather than why they do not, and there has been very little research into how policy affects dog-walking behaviour.

The purpose of this review was to provide information on correlates that may be useful for the design of interventions to encourage dog walking. However, intervention studies need to measure dog walking in the context of overall physical activity, in order to determine whether the intervention increases the physical activity of those who are already physically active, or changes the environment in which physical activity occurs. Although encouraging those owners who already walk their dogs regularly to walk even more is not problematic, the priority for improving population health should be to increase the physical activity of those owners who currently do not undertake physical activity on a regular basis. Intervention studies must also openly report the tool used to deliver the intervention, designed with our model of specific behavioural correlates in mind so that these can be clearly identified and their effects measured. For example it is difficult to evaluate exactly how giving ‘persuasive material about canine health from walking’ [41] is acting on owner’s dog walking behaviour: which particular aspects of canine health is it describing benefits to? (physical? mental?); is it changing perceived exercise requirements?; is it increasing the owner’s overall perception of the valuation of exercise for dog?; is it increasing the sense of dog obligation?; or even using the subjective norm of significant others depending on how the intervention is delivered (e.g.; veterinarians)?

Finally, this review has prioritised the individual health impacts that may be gained by encouraging a person to walk more with their dog. However, the positive impacts of dog owners walking their dogs may also extend to non-owners, through an increased sense of safety in the neighbourhood (see [88]) as well as sense of community and social capital [74]. It must also be acknowledged that a few studies have reported negative impacts of dog walking or dogs as a barrier to physical activity, for example through concerns about loose/stray dogs and dog waste [88–90] or being an injury hazard through bites or falls [91–94]. There are also concerns over the impact of dog walking on wildlife [53, 95–100]. Any attempts to promote dog walking activity must be done in a manner that is also mindful to the potential negative impacts of dog walking on society; and any attempts to prevent or reduce dog walking and its associated impacts should also be aware of the negative effect this may have on the health of dogs and their owners.

## Conclusion

Current evidence suggests that dog walking may be most effectively encouraged through targeting the dog-owner relationship and by providing dog-supportive physical environments. More research is required to investigate the influence of individual owner and dog factors on ‘intention’ to walk the dog as well as the influence of human social interaction whilst walking a dog. The effects of policy and cultural practices relating to dog ownership and walking should also be investigated. Future studies must account for the effects of confounding between variables, and preferably use longitudinal designs or where possible test the effectiveness of identified correlates using interventions with a controlled design, in order to infer causality.
